# Internal Validation of Mitochondrial DNA Control Region Using the Precision ID mtDNA Control Region Panel

**DOI:** 10.3390/genes16121504

**Published:** 2025-12-16

**Authors:** Esther Lechuga-Morillas, María Saiz, Diana C. Vinueza-Espinosa, Xiomara Gálvez, María Isabel Medina-Lozano, Rosario Medina-Lozano, Francisco Santisteban, Juan Carlos Álvarez, José Antonio Lorente

**Affiliations:** Laboratory of Genetic Identification & Human Rights (LABIGEN-UGR), Department of Legal Medicine, Faculty of Medicine, University of Granada, PTS Granada, Av. Investigación 11, 18016 Granada, Spain

**Keywords:** NGS, forensic genetics, mitochondrial DNA, massive parallel sequencing

## Abstract

**Background/Objectives:** The sequencing of mitochondrial DNA is a valuable tool in forensic genetics, particularly in cases involving degraded samples or those with low nuclear DNA content. In this study, we performed an internal validation for an NGS-based typing of the mitochondrial DNA control region using the Precision ID mtDNA Control Region Panel on the Ion S5^TM^ sequencer (Thermo Fisher Scientific, Waltham, MA, USA). This validation enhances the scientific robustness, reliability, and judicial admissibility of the results in forensic cases. **Methods:** Six parameters were evaluated: minimum read depth, sensitivity, repeatability, concordance with Sanger, reproducibility and heteroplasmy detection employing ten negative controls, nine reference samples, a bone sample, and six experimental mixtures. Libraries were prepared using the Ion Chef^TM^ system, quantified on the Quantstudio^TM^ 5 Real-Time PCR, sequenced on the Ion GeneStudio^TM^ S5, and analyzed with Converge^TM^ software. **Results:** In this study, we found that a read depth threshold of 100 reads per position, an optimal concentration of 20 pg/µL, and a detection threshold of heteroplasmies of 20% are appropriate to obtain reliable genetic profiles. This supports the application of this method in forensic casework, in which initial concentrations may be around the optimal concentration exposed here due to the provenience of the samples. **Conclusions:** The results indicate that the NGS platform is suitable for forensic mtDNA analysis, even under low-template conditions, and offers higher sensitivity compared to Sanger sequencing. However, some limitations were observed in the coverage of specific amplicons, the detections of polymorphisms in homopolymeric regions, and in the detection of low-level heteroplasmies.

## 1. Introduction

Forensic genetics plays a crucial role in human identification, kinship testing, and criminal investigations. Conventional approaches rely heavily on nuclear DNA markers such as Short Tandem Repeats (STRs). However, in many forensic scenarios—such as mass disasters or historical remains—nuclear DNA is often degraded or insufficient, limiting its usefulness. Due to its high copy number per cell, lack of recombination, maternal inheritance, high mutation rate, and greater resistance to degradation, mtDNA provides valuable genetic information that complements nuclear DNA studies [1].

The control region of the mitochondrial genome, also referred to as the D-loop, contains three hypervariable regions (HV-I, HV-II, HV-III) that harbor a high density of polymorphisms [2]. These regions are widely used for forensic purposes, as they enable the establishment of maternal lineages and facilitate the comparison of profiles with population databases. Despite its advantages, mtDNA analysis also presents limitations: its haploid nature restricts discrimination power among maternal relatives, and the relatively high mutation rate may result in heteroplasmic states—where more than one mtDNA variant coexists within an individual [3].

Historically, mtDNA sequencing was conducted using Sanger sequencing, which provided reliable results but had important limitations in sensitivity, throughput, and the detection of low-level heteroplasmies [4]. The developmental of massively parallel sequencing, also known as next-generation sequencing (NGS), revolutionized forensic genetics by enabling high-resolution mtDNA analysis with increased sensitivity, scalability, and the ability to simultaneously process multiple samples [5,6,7].

Currently, the most widely used NGS platforms in forensic laboratories are MiSeq FGX and Ion Torrent. Both have been integrated with specialized typing kits and bioinformatics tools that enable the sequencing and analysis of the mtDNA control region. However, before incorporating different kits into routine forensic workflows, laboratories must validate their performance internally to ensure compliance with international standards, and to verify their robustness under casework-like conditions [8].

International organizations, including the International Society for Forensic Genetics (ISFG) [9], the Scientific Working Group on DNA Analysis Methods (SWGDAM) [10], and the European Network of Forensic Science Institutes (ENFSI) [11], have issued guidelines for validation studies, emphasizing the evaluation of parameters such as sensitivity, reproducibility, concordance, and heteroplasmy detection. Furthermore, the ISO/IEC 17025:2017 standard establishes the traceability and reliability of analytical results [12].

In this work, we describe an internal validation study of the NGS-based typing of the mtDNA control region, conducted using the Precision ID mtDNA Control Region Panel on the Ion S5^TM^ System (Thermo Fisher Scientific, Waltham, MA, USA). The aim of this study was to evaluate the analytical performance of this panel by assessing key parameters under conditions that simulate routine forensic casework. This validation seeks to provide evidence of the panel’s reliability and robustness prior to its implementation in forensic laboratories, generating independent validation data that have not been reported beyond those provided by the manufacturer.

## 2. Materials and Methods

### 2.1. Validation Parameters and Sample Description

The selected samples, analyzed at LABIGÉN (Granada, Spain), were divided into a series of experiments in order to assess minimum read depth, sensitivity, repeatability, concordance, reproducibility, and heteroplasmy detection limit. The number of samples was defined according to the ISFG and SWGDAM criteria for internal validation studies, prioritizing the representativeness of sample types commonly encountered in real casework (reference samples, degraded bone samples, and mixtures).

In addition, several analytical parameters were defined to ensure consistent data interpretation: coverage, referring to the positions of the mtDNA control region successfully sequenced by the Ion S5^TM^; total read depth, representing the number of forward and reverse reads obtained per position (i.e., how many time each position was sequenced); and total allele read depth, defined as the number of reads supporting a specific polymorphism at a given position in both forward and reverse directions.

Minimum read depth was evaluated using four aliquots of amplification-grade water, three negative controls from the DNA extraction of reference samples, and three negative controls from the DNA extraction of bone samples.

For sensitivity and repeatability studies, 4 serial dilutions were manually prepared in amplification-grade water (Promega Corporation, Madison, WI, USA) using the C007 control DNA, a reference sample (RS1), and a human remain (HR1), and examined in triplicate at the final genomic DNA (gDNA) input of 20 pg/µL (C1), 5 pg/µL (C2), 1 pg/µL (C3), and 0.25 pg/µL (C4), which were processed in independent runs for each sample.

The concordance study was performed by comparing Sanger data generated using the ABI3500 Genetic Analyzer (Thermo Fisher Scientific, Waltham, MA, USA) with NGS data produced with the Ion S5^TM^ system (Thermo Fisher Scientific, Waltham, MA, USA). Four single-source samples from buccal swabs (RS4, RS5, RS6, and RS7) obtained from voluntary adults following written informed consent were analyzed at LABIGÉN.

The reproducibility analysis consisted of the comparison of NGS data produced with the Ion S5^TM^ System (Thermo Fisher Scientific, Waltham, MA, USA) at LABIGÉN by two different analysts. Four single-source samples from buccal swabs (RS4, RS5, RS8, and RS9) obtained from voluntary adults following written informed consent were analyzed.

To establish the detection threshold for heteroplasmy, mixtures of two DNA reference samples were sequenced in varying theoretical ratios: M1 (50:50), M2 (40:60), M3 (30:70), M4 (20:80), M5 (10:90), and M6 (5:95).

The minimum read depth threshold was estimated as the average of the maximum read depths observed across the analyzed samples. For the sensitivity study, a descriptive analysis and an ANOVA with post hoc testing of the total allele read depth were performed for the expected polymorphisms, which had been previously obtained by Sanger sequencing. In contrast, repeatability was evaluated through descriptive statistics and an ANOVA with post hoc testing of both total read depth and total allele read depth for the observed polymorphisms, referring to those detected by the Ion S5^TM^. Finally, reproducibility was assessed by performing descriptive analyses of the polymorphisms observed in independent sequencing runs conducted by two analysts using the same instrument under similar experimental conditions. These tests were performed using Jamovi Desktop Software v.2.6.44.

### 2.2. DNA Extraction and Quantification

Genomic DNA was isolated from buccal swab samples using the QIAamp DNA Investigator Kit on an QIAcube (QIAGEN, Hilden, Germany) according to the manufacturer’s instructions. The bone sample was pretreated before DNA extraction. The sample’s exterior surface was sanded and then cut into 0.5–1 cm fragments with a Dremel ^®^ rotatory tool (Dremel, Mount Prospect, IL, USA). After that, fragments were exposed to UV light in a 6 W UV cabin, for 10 min each side, and then ground in a TissueLyser II (QIAGEN, Hilden, Germany) under two cycles of 30 Hz for 30 s. Three replicates of 200 mg each of bone powder were used to extract DNA using the PrepFiler BTA Express Kit on an AutoMate Express^TM^ Forensic DNA Extraction System (Thermo Fisher Scientific, Waltham, MA, USA) following the recommended protocol. DNA yield from reference samples was quantified using the Qubit^TM^ 1X dsDNA HS Kit on a Qubit 4 Flex (Invitrogen, Waltham, MA, USA), whereas bone-derived DNA was quantified in duplicate with the Quantifiler^TM^ Trio DNA Quantification Kit on the Quantstudio^TM^ 5 Real-Time PCR (Thermo Fisher Scientific, Waltham, MA, USA) and analyzed using HID Real-Time PCR Analysis Software v.1.4 (Thermo Fisher Scientific, Waltham, MA, USA). All DNA extracts were subsequently diluted in amplification-grade water to a final concentration of 20 pg/µL, except for the sensitivity experiments, in which serial dilutions were prepared as described above.

### 2.3. Library and Template Preparation

**Figure 1:**
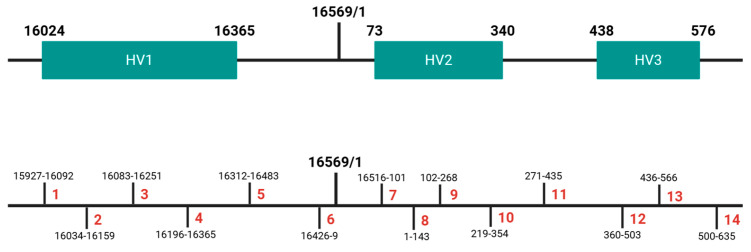
Representation of the primer pairs’ arrangement.

Library construction was carried out with the Precision ID mtDNA Control Region Panel and the Precision ID DL8 Kit (Thermo Fisher Scientific, Waltham, MA, USA) using the Ion Chef™ System. The panel, designed specifically for forensic use, employs a two-pool AmpliSeq multiplex strategy comprising seven primer pairs per pool plus several degenerated primer pairs [13]. The range of the different amplicons are shown in Figure 1.

Negative controls included in the minimum read depth assessment were processed under identical conditions to the test samples to monitor potential contamination, while the C007 control DNA served as a positive control for amplification and library preparation.

Following primer digestion and adapter ligation, the libraries were quantified with the Ion Library TaqMan™ Quantitation Kit (Thermo Fisher Scientific, Waltham, MA, USA) according to the manufacturer’s protocol, and normalized to 30 pM to ensure equal representation in pooled libraries. Barcoded libraries were combined using 12 µL of each. Automated template preparation, enrichment of template-positive beads, and chip loading were performed on the Ion Chef™ System with the Ion S5™ Precision ID Chef Kit, following the guidelines in the Thermo Fisher Scientific application manual for mtDNA panels on the HID Ion S5™/HID Ion GeneStudio™ S5 System [13].

### 2.4. Sequencing and Data Analysis

All samples in this study were sequenced across four runs on the Ion S5™ System using the Ion S5™ Precision ID Sequencing Kit and Ion 520™ chips (Thermo Fisher Scientific, Waltham, MA, USA), following the manufacturer’s guidelines. Raw sequencing data were generated with Ion Torrent Suite™ Software v.5.12.3 (Thermo Fisher Scientific, Waltham, MA, USA) and aligned to the revised Cambridge Reference Sequence (rCRS +80; NCBI reference NC_012920), as recommended by Thermo Fisher Scientific, using default alignment parameters. Secondary data analysis was carried out using the HID Genotyper plug-in in Converge^TM^ Software v.2.2 (Thermo Fisher Scientific, Waltham, MA, USA). Converge employs the ‘mito variant caller’ (MVC), an optimized alignment algorithm that incorporates PhyloTree mtDNA phylogeny and EMPOP database information into its scoring system [14]. MVC parameters were set to a minimum of 20 total reads per position, a minimum of 20 variant reads for calling, a 20-read threshold to flag low-coverage regions, and a minimum coverage percentage of 5.0 relative to the amplicon median. Additional thresholds included a score of 96.0 for variant confirmation, 10.0 for point heteroplasmy, 20.0 for insertions, and 30.0 for deletions. Variant calls, coverage metrics, and quality scores were automatically reported by Converge in both tabular summaries, and linear–circular graphical plots.

Sanger sequencing data were obtained using the BigDye^TM^ Terminator V.1.1 Cycle Sequencing kit on the ABI3500 Genetic Analyzer and analyzed with GeneMapper^TM^ ID-X Software v.1.4 (Thermo Fisher Scientific, Waltham, MA, USA).

## 3. Results and Discussion

### 3.1. Analysis of Minimum Read Depth

To assess the minimum read depth in this study, we analyzed the results from four aliquots of the water used in the dilution steps and six extraction negative controls sequenced throughout the study.

None of these samples provided a complete mtDNA sequence, nor usable data for comparison; however, all of them exhibited several amplicons covered by aligned reads with a local maximum read depth ranging from 14 to 234 (Table 1). Despite the origin of these reads being unclear, they may arise at least from very low levels of contaminating DNA or the nature of the selected samples. Regardless, these reads seemed shorter than all of the amplicons of the Precision ID mtDNA Control Region Panel, whose average size range is 153 bp.

Based on the average of the maximum read depths of the negative controls, a minimum total read depth of 100 reads per position was established as the read depth threshold to ensure reliable base calling and consistent variant interpretation. All subsequent samples were reanalyzed applying this criterion.

This threshold is substantially higher than that reported in previous studies, where a minimum read depth of 20 reads per position was deemed acceptable [15,16,17]. However, our findings are in line with the recommendations of Brandhagen et al. [18], who likewise advocate for a minimum read depth of 100 reads as a criterion for the reliable interpretation of mitochondrial DNA sequences.

### 3.2. Sensitivity and Repeatability Assessment

The sensitivity of the Precision ID mtDNA Control Region Panel was evaluated using three types of samples: the C007 control DNA, a reference sample, and a bone sample, with different amounts of gDNA ranging from 20 to 0.25 pg/µL. Meanwhile, a repeatability assessment was performed with the processing of three independent replicates for each concentration. Each replicate underwent separate library preparation with distinct barcodes and was sequenced on different days. Genetic profiles were compared against reference profiles for samples C007, RS1, and HR1 to estimate potential information loss.

Full coverage (100%) of the mtDNA control region (positions 16024-576) was achieved for all sample replicates. Significant differences (*p*-value < 0.001) in total read depth were observed between the commercial control DNA and both samples (RS1 and HR1), attributable to intrinsic differences in sample quality, as the manufacturer-provided control typically exhibits higher integrity than buccal swabs or bone remains. However, no significant differences were detected between the total real depth values of RS1 and HR1 (*p*-value = 0.032). Likewise, no significant differences were observed in total allele read depth when comparing high-quality DNA with bone-derived DNA profiles (*p*-value = 0.297).

In contrast, DNA concentration had a significant impact on total allele read depth (*p*-value < 0.001). A progressive decrease in mean allele read depth with decreasing DNA concentration was observed, as shown in Supplementary Table S1. Significant differences were also detected across concentrations in both the total read depth (*p*-value < 0.001) and recovery of expected polymorphisms (*p*-value = 0.003).

The C007 sample yielded 96% recovery of the expected polymorphisms across all tested concentrations relative to the manufacturer’s reference dataset. RS1 achieved complete recovery (100%) of expected polymorphisms—previously obtained and validated by Sanger sequencing—whereas the bone-derived sample HR1 showed a 80.5% recovery rate, with partial profiles at lower DNA inputs. The remaining 20% corresponded either to undetected polymorphisms or to variants incorrectly classified as heteroplasmies, likely to degradation-related effects that intensified with decreasing DNA concentration. These results are illustrated as heatmaps in Supplementary Tables S2–S4. However, no significant differences in total allele read depth were observed among replicates at each concentration, as shown in Supplementary Tables S5–S10.

Nevertheless, in C007, a deletion at position 309 and a length heteroplasmy at position 573 (573.4C) were detected, which were not reported by the manufacturer due to lack of coverage in that region. Previous studies, such as Cihlar et al. (2020) [19], reported the 573.4C polymorphism in several C007 lots, although deletions at position 309—observed in one of the C2 replicates and in the third replicate of C3—were not detected. However, based on the EMPOP and MITOMAP data [20,21,22], which indicates that 309del is a rare variant, these 309del calls were considered false deletions.

In RS1, artifacts appeared from concentration C2 and increased at lower inputs. However, 100% of expected polymorphisms were recovered within the tested range. In HR1, the degraded nature of bone DNA led to progressively more artifacts with decreasing DNA concentration, resulting in increased background noise, reduced sequencing efficiency, and variant-calling errors, consistent with the findings of Senst et al. [23]. Detailed results are provided in Supplementary Tables S12–S14.

Accordingly, decreases in total read depth and allele read depth at input concentration below 20 pg/µL are evident.

**Figure 2:**
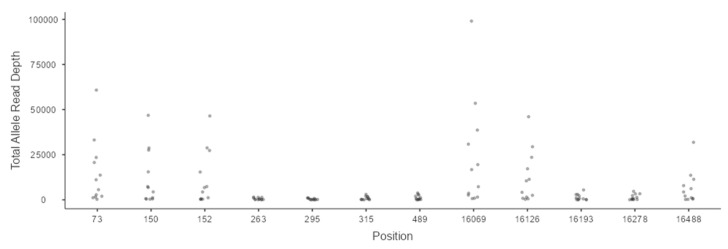
Jitter plot showing the total allele read depth of the expected polymorphisms across all concentrations and replicates for sample HR1.

It is important to note that the occurrence of artifacts and loss of expected polymorphisms is not solely tied to DNA quantity; certain amplicons (3, 10, 11, and 14) exhibit suboptimal design, spanning homopolymeric poly-C regions (Figure 2 and Supplementary Figures S1 and S2). These regions inherently produce lower read depth and unreliable variant calls, requiring careful analyst review and, when necessary, confirmation with an alternative sequencing chemistry.

Overall, our findings suggest that a DNA input of 20 pg/µL constitutes an optimal threshold for generating complete and reliable NGS profiles from both reference and bone samples. Although full profiles can occasionally be obtained at lower concentrations, stochastic effects and sequencing artifacts increase substantially, complicating result interpretation. These observations are consistent with Faccinetto et al., who reported complete profiles from 15 pg, with lower inputs associated with allelic dropout, false positives, and loss of expected information [17]. Reported thresholds in the literature vary, with complete profiles observed from 4 pg to 50 pg [16,24,25].

### 3.3. Concordance Study

Concordance was evaluated by comparing the control region mtDNA sequences of four reference samples (RS4–RS7) obtained via Sanger sequencing by a laboratory researcher with the corresponding sequences generated using the Ion S5 NGS platform. This comparison aimed to assess the accuracy and reliability of the NGS-based method relative to the established Sanger sequencing standard. The results are summarized in Table 2.

Sample RS4 showed 83% concordance, with the sole discrepancy being a length heteroplasmy at position 309. This difference likely reflects the inherent complexity of detecting variants in cytosine-rich hypervariable regions, which pose challenges for both sequencing and alignment. For RS5, concordance was 92.8%, with a single discrepancy corresponding to a length heteroplasmy at position 573. This may be attributable to low coverage in Sanger sequencing, differences in sensitivity between the two methods, or the chemical characteristics of homopolymeric regions, which complicate interpretation. Although this variant is common in the population [26], accurate detection requires careful analysis, highlighting the advantage of NGS in identifying low-frequency heteroplasmies often missed by Sanger sequencing.

In contrast, RS6 and RS7 exhibited complete concordance (100%) between NGS and Sanger sequencing, consistent with previous reports demonstrating high consistency between these methods for reference profiles [17,23,27,28,29].

Overall, these findings support the validity of NGS as a highly sensitive and precise tool for detecting mitochondrial variants, particularly in hypervariable regions, and for low-frequency heteroplasmies, reinforcing its role as a complementary—or, in certain forensic contexts, superior—approach to conventional Sanger sequencing.

### 3.4. Reproducibility Analysis

Reproducibility of the Precision ID mtDNA Control Region Panel was evaluated by comparing the sequencing results of four reference samples (RS4, RS5, RS8, and RS9) independently by two different analysts using the same Ion S5^TM^ platform and protocol. This approach enables the evaluation of inter-analyst consistency, an essential factor in forensic applications.

When excluding polymorphisms located within regions covered by amplicons 3 (16083-16251), 10 (219-354), 11 (271-435), and 14 (500-635)—all of which showed reduced read depth—the samples exhibited 100% reproducibility. Nonetheless, Supplementary Table S15 provides a complete list of the polymorphisms detected in both sequencing runs, including those located in the low-coverage amplicons, alongside the corresponding concordance values. Notably, all observed discrepancies were attributable to insufficient read depth in cytosin-rich homopolymeric regions, which are well known to be prone to sequencing and alignment artifacts [26]. These inconsistencies likely arose from technical factors such as read depth and alignment performance.

Overall, discrepancies were concentrated in homopolymeric or low-read-depth regions, highlighting the importance of manual review to ensure error-free profiles. Despite these localized issues, the method achieved full reproducibility and aligns with previous reports [17,18,23,30].

Collectively, these results highlight the robustness of the method while emphasizing the need for the careful interpretation of variants in technically challenging genomic regions, particularly in high-stakes forensic contexts.

### 3.5. Heteroplasmy

Point heteroplasmy detection was assessed by generating a mixture from two reference samples. The selected samples exhibited multiple sequence variants relative to the reference genome, generating eight heteroplasmic sites within the mitochondrial control region, including polymorphisms frequently reported in the Spanish population such as 152Y, 295Y, and 16093Y, previously described by Santos et al. [31]. In addition, a commercial standard sample (DNA mix; Promega Corporation, Madison, WI, USA), consisting of equimolar nuclear DNA from one male and one female donor, was analyzed.

The results, summarized in Table 3, revealed imbalances between observed and expected allelic proportions, consistent with the findings of Churchill et al. [32], who also reported the underrepresentation of minor alleles under certain experimental conditions. In addition, minor contributors may appear with variant frequencies comparable to, or even exceeding, those of the major contributor, a phenomenon also observed by Cihlar et al. [15]. Such variability may arise from differences in amplification efficiency, library preparation, or variation in mitochondrial copy numbers between samples [15,33].

Significant allelic imbalances can compromise the detection of true heteroplasmies, particularly when the minor allele frequency falls below 10% [15,18,30]. For this reason, an operational heteroplasmy detection threshold of 20% was established in this study, with allelic ratios of 80:20 or greater considered reliable for forensic interpretation.

Based on the established threshold, the analysis of the DNA mix sample revealed unequal contributions of each profile despite the theoretical 50:50 ratio (shown in Supplementary Table S11). This finding indicates that estimating allelic proportions solely from nuclear DNA quantification is unreliable, as mitochondrial DNA copy number does not maintain a linear or constant relationship with nuclear DNA content [3]. These results highlight the necessity of specific mitochondrial DNA quantification to ensure the accurate interpretation of allelic proportions in forensic analysis. In addition, technical biases arising from mixture preparation, library construction, and highly variable genomic regions—particularly positions 16184-16193 and 303-315—should be carefully considered, as their interpretation can be especially challenging [31].

## 4. Conclusions

This study provides an internal validation of the Precision ID mtDNA Control Region Panel performed on the Ion S5™ platform using the C007 control DNA, DNA mix, and representative forensic specimens. The method showed 100% sensitivity at 20 pg/µL, and a minimum read depth of 100 reads per position was established to ensure accurate variant calling and consistent interpretation. Repeatability was shown to be 100% across all tested concentrations, with no statistically significant differences between replicates, confirming the stability of the workflow under intra-analyst conditions.

The results showed an average concordance of 93.95% with Sanger sequencing, with values ranging from 83% to 100% depending on the sample, and 100% reproducibility between analysts except for discrepancies confined to cytosine-rich homopolymeric regions, which are known to challenge MPS chemistries. The panel showed robust detections of expected variants and reliable heteroplasmy identification at a 20% operational threshold, illustrating its enhanced resolution compared with conventional sequencing. Although certain amplicons exhibited reduced performance, the overall robustness of the method supports its suitability for forensic casework involving both high-quality and compromised samples.

Overall, these results support the implementation of this MPS workflow in forensic laboratories. Future work should include interlaboratory comparison studies and expanded evaluation using highly degraded samples to strengthen external validations and further consolidate its forensic applicability.

## Figures and Tables

**Table 1:** Average of maximum read depth.

Sample	Maximum Read Depth
H_2_O 1	96
H_2_O 2	14
H_2_O 3	39
H_2_O 4	119
Ctrl- 1 RS	170
Ctrl- 2 RS	234
Ctrl- 3 RS	158
Ctrl- 1 HR	66
Ctrl- 2 HR	55
Ctrl- 3 HR	56
Average	100.7

**Table 2:** Sequences obtained via Sanger sequencing vs. NGS.

Sample	Concordance	Method	Sequence
RS4	83%	Sanger	263G, 309.2C *, 315.1C, 456T, 16304C, 16519C
		NGS	263G, 309.1C *, 315.1C, 456T, 16304C, 16519C
RS5	92.8%	Sanger	73G, 152C, 199C, 250C, 263G, 309.1C, 315.1C, 494A, 16129A, 16148T, 16223T, 16391A, 16519C
		NGS	73G, 152C, 199C, 250C, 263G, 309.1C, 315.1C, 494A, 573.5C *, 16129A, 16148T, 16223T, 16391A, 16519C
RS6	100%	Sanger	73G, 151T, 152C, 182T, 186A, 189C, 195C, 198T, 204C, 207A, 247del, 263G, 297G, 315.1C, 316A, 523del, 524del, 16037G, 16129A, 16187T, 16189C, 16223T, 16243C, 16278T, 16293G, 16294T, 16311C, 16360T, 16519C
		NGS	73G, 151T, 152C, 182T, 186A, 189C, 195C, 198T, 204C, 207A, 247del, 263G, 297G, 315.1C, 316A, 523del, 524del, 16037G, 16129A, 16187T, 16189C, 16223T, 16243C, 16278T, 16293G, 16294T, 16311C, 16360T, 16519C
RS7	100%	Sanger	73G, 151T, 152C, 182T, 186A, 189C, 195C, 198T, 204C, 207A, 247del, 263G, 297G, 315.1C, 316A, 523del, 524del, 16037G, 16129A, 16187T, 16189C, 16223T, 16243C, 16278T, 16293G, 16294T, 16311C, 16360T, 16519C
		NGS	73G, 151T, 152C, 182T, 186A, 189C, 195C, 198T, 204C, 207A, 247del, 263G, 297G, 315.1C, 316A, 523del, 524del, 16037G, 16129A, 16187T, 16189C, 16223T, 16243C, 16278T, 16293G, 16294T, 16311C, 16360T, 16519C

* Discrepancies between Sanger sequencing and NGS.

**Table 3:** Empirically observed allelic proportions in heteroplasmic mixtures (M1–M6).

Sample	Polymorphisms (minor:major)	M1 (%:%)	M2 (%:%)	M3 (%:%)	M4 (%:%)	M5 (%:%)	M6 (%:%)
Het1	152 (C:T)	61:33	52:42	43:52	31:66	17:81	14:84
	295 (T:C)	57:40	46:51	38:56	34:62	21:75	17:78
	462 (T:C)	79:20	67:33	59:41	44:56	27:73	23:77
	489 (C:T)	78:21	63:36	54:45	43:57	23:78	21:79
	16069 (T:C)	70:30	60:40	48:52	34:66	19:81	15:85
	16093 (T:C)	75:25	65:35	55:45	41:59	27:73	24:76
	16126 (C:T)	72:27	63:37	52:48	37:63	21:79	18:82
	16193 (T:C)	72:25	64:32	50:45	36:62	22:76	18:79

## Data Availability

All data is publicly available except for the internal calculations/statistics.

## Supplementary Materials

The following supporting information can be downloaded at https://www.mdpi.com/article/10.3390/genes16121504/s1. Table S1: Mean allele read depth values; Table S2: Heatmap C007. In green, the correct reading of the expected polymorphisms present in this genetic profile can be observed; in red, those alleles that have not been identified as variations from the reference sequence due to low coverage of the region; Table S3: Heatmap RS1. In green, the correct reading of the expected polymorphisms present in this genetic profile can be observed; Table S4: Heatmap HR1. In green, the correct reading of the expected polymorphisms present in this genetic profile can be observed; in red, those alleles that have not been identified as variations from the reference sequence due to low coverage of the region, and polymorphisms that have not been correctly determined are shown in blue; Table S5: *p*-values of total allele read depth for C1 and C2 of C007; Table S6: *p*-values of total allele read depth for C3 and C4 of C007; Table S7: *p*-values of total allele read depth for C1 and C2 of RS1; Table S8: *p*-values of total allele read depth for C3 and C4 of RS1; Table S9: *p*-values of total allele read depth for C1 and C2 of HR1; Table S10: *p*-values of total allele read depth for C3 and C4 of HR1; Table S11: Theoretical and empirical percentages of allelic proportions in DNA mix; Table S12: C007 sequences obtained through NGS. Artifacts generated by the equipment during the method are shown in orange, a length heteroplasmy not reported by the manufacturer is shown in blue, and positions whose polymorphisms were not detected during sequencing are shown in red; Table S13: RS1 sequences obtained through NGS. Artifacts generated by the equipment during the method are shown in orange; Table S14: HR1 sequences obtained through NGS. Artifacts generated by the equipment during the method are shown in orange, polymorphisms that have not been correctly determined are shown in blue, and positions whose polymorphisms were not detected during sequencing are shown in red; Table S15: Sequences obtained via NGS; Figure S1: Jitter plot showing the total allele read depth of the expected polymorphisms across all concentrations and replicates for sample C007; Figure S2: Jitter plot showing the total allele read depth of the expected polymorphisms across all concentrations and replicates for sample RS1.
