# Supplementary material for: Internal Validation of Mitochondrial DNA Control Region Using the Precision ID mtDNA Control Region Panel
Source: Genes (Basel). 2025 Dec 16;16(12):1504. doi: 10.3390/genes16121504 (PMC12732821; doi:10.3390/genes16121504)
Supplement: Supplementary file 1 [file genes-16-01504-s001.zip › genes-3970115-supplementary.pdf]

**Table S1:** Mean allele read depth values.

| | Concentration | | | |
|---|---|---|---|---|
| **Sample** | **C1 (20 pg/ µL)** | **C2 (5 pg/ µL)** | **C3 (1 pg/ µL)** | **C4 (0.25 pg/ µL)** |
| **C007** | 8625 | 6278 | 5711 | 869 |
| **RS1** | 11002 | 7370 | 4298 | 1406 |
| **HR1** | 17598 | 8121 | 2528 | 357 |

**Table S2:** Heatmap C007. In green, the correct reading of the expected polymorphisms present in this genetic profile can be observed; in red, those alleles that have not been identified as variations from the reference sequence due to low coverage of the region.

| | **C007** | | | | | | | | | | | |
|---|---|---|---|---|---|---|---|---|---|---|---|---|
| | **C1 (20 pg/ µL)** | | | **C2 (5 pg/ µL)** | | | **C3 (1 pg/ µL)** | | | **C4 (0.25 pg/ µL)** | | |
| | **R1** | **R2** | **R3** | **R1** | **R2** | **R3** | **R1** | **R2** | **R3** | **R1** | **R2** | **R3** |
| **73G** | | | | | | | | | | | | |
| **152C** | | | | | | | | | | | | |
| **199C** | | | | | | | | | | | | |
| **204C** | | | | | | | | | | | | |
| **207A** | | | | | | | | | | | | |
| **250C** | | | | | | | | | | | | |
| **263G** | | | | | | | | | | | | |
| **315.1C** | | | | | | | | | | | | |
| **460C** | | | | | | | | | | | | |
| **16129A** | | | | | | | | | | | | |
| **16223T** | | | | | | | | | | | | |
| **16391A** | | | | | | | | | | | | |
| **16519C** | | | | | | | | | | | | |

**Table S3:** Heatmap RS1. In green, the correct reading of the expected polymorphisms present in this genetic profile can be observed.

| | **RS1** | | | | | | | | | | | |
|---|---|---|---|---|---|---|---|---|---|---|---|---|
| | **C1 (20 pg/ µL)** | | | **C2 (5 pg/ µL)** | | | **C3 (1 pg/ µL)** | | | **C4 (0.25 pg/ µL)** | | |
| | **R1** | **R2** | **R3** | **R1** | **R2** | **R3** | **R1** | **R2** | **R3** | **R1** | **R2** | **R3** |
| **73G** | | | | | | | | | | | | |
| **150T** | | | | | | | | | | | | |
| **152C** | | | | | | | | | | | | |
| **263G** | | | | | | | | | | | | |
| **295T** | | | | | | | | | | | | |
| **315.1C** | | | | | | | | | | | | |
| **489C** | | | | | | | | | | | | |
| **16069T** | | | | | | | | | | | | |
| **16126C** | | | | | | | | | | | | |
| **16193T** | | | | | | | | | | | | |
| **16278T** | | | | | | | | | | | | |

**Table S4:** Heatmap HR1. In green, the correct reading of the expected polymorphisms present in this genetic profile can be observed; in red, those alleles that have not been identified as variations from the reference sequence due to low coverage of the region, and polymorphisms that have not been correctly determined are shown in blue.

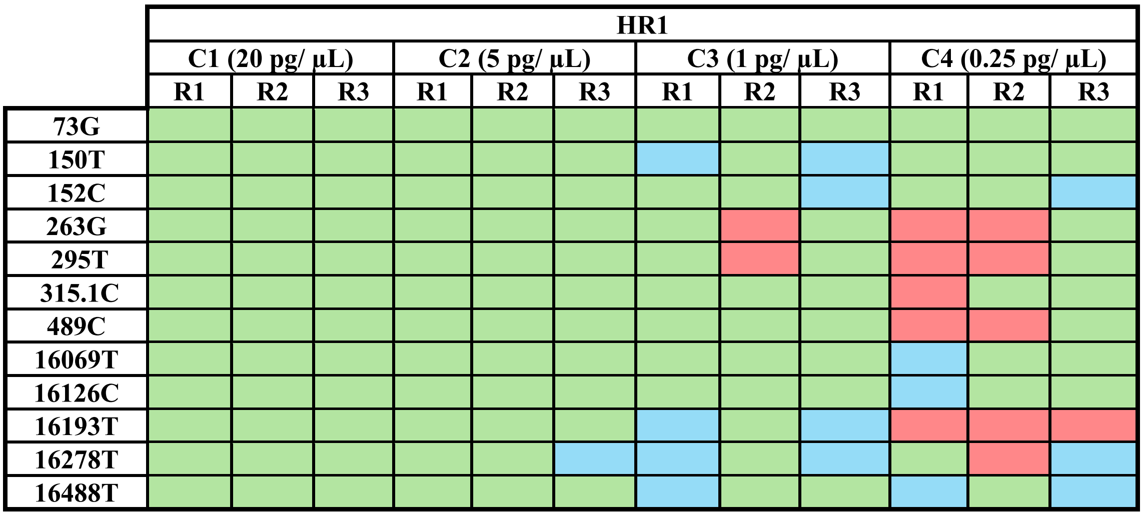


**Table S5:** P-values of total allele read depth for C1 and C2 of C007.

| C007 | R1 | R2 | R3 | |
|---|---|---|---|---|
| R1 | 0 | 0.432 | 0.086 | C1 (20 pg/ µL) |
| R2 | 1 | 0 | 1 | |
| R3 | 0.094 | 0.174 | 0 | |
| | C2 (5 pg/ µL) | | | |

**Table S6:** P-values of total allele read depth for C3 and C4 of C007.

| C007 | R1 | R2 | R3 | |
|---|---|---|---|---|
| R1 | 0 | 1 | 0.346 | C3 (1 pg/ µL) |
| R2 | 0.926 | 0 | 0.149 | |
| R3 | 1 | 0.644 | 0 | |
| | C4 (0.25 pg/ µL) | | | |

**Table S7:** P-values of total allele read depth for C1 and C2 of RS1.

| RS1 | R1 | R2 | R3 | |
|---|---|---|---|---|
| R1 | 0 | 1 | 0.564 | C1 (20 pg/ µL) |
| R2 | 0.59 | 0 | 0.752 | |
| R3 | 1 | 0.193 | 0 | |
| | C2 (5 pg/ µL) | | | |

**Table S8:** P-values of total allele read depth for C3 and C4 of RS1.

| RS1 | R1 | R2 | R3 | |
|---|---|---|---|---|
| R1 | 0 | 1 | 1 | C3 (1 pg/ µL) |
| R2 | 0.153 | 0 | 1 | |
| R3 | 1 | 0.403 | 0 | |
| | C4 (0.25 pg/ µL) | | | |

**Table S9:** P-values of total allele read depth for C1 and C2 of HR1.

| HR1 | R1 | R2 | R3 | |
|---|---|---|---|---|
| **R1** | 0 | 0.081 | 0.222 | **C1 (20 pg/ µL)** |
| **R2** | 0.008 | 0 | 1 | |
| **R3** | 0.035 | 1 | 0 | |
| | **C2 (5 pg/ µL)** | | | |

**Table S10:** P-values of total allele read depth for C3 and C4 of HR1.

| HR1 | R1 | R2 | R3 | |
|---|---|---|---|---|
| **R1** | 0 | 0.007 | 0.014 | **C3 (1 pg/ µL)** |
| **R2** | 0.798 | 0 | 1 | |
| **R3** | 1 | 0.424 | 0 | |
| | **C4 (0.25 pg/ µL)** | | | |

**Table S11:** Theoretical and empirical percentages of allelic proportions in DNA mix.

| | DNA mix | |
|---|---|---|
| **Positions** | **Theoretical percentage (minor:major)** | **Empirical percentage (minor:major)** |
| **150 (T:C)** | 50:50 | 34:66 |
| **195 (C:T)** | 50:50 | 27:73 |
| **279 (C:T)** | 50:50 | 21:79 |
| **16192 (T:C)** | 50:50 | 24:75 |
| **16223 (T:C)** | 50:50 | 29:71 |
| **16270 (T:C)** | 50:50 | 21:79 |
| **16292 (T:C)** | 50:50 | 21:78 |
| **16519 (T:C)** | 50:50 | 35:64 |

**Table S12:** C007 sequences obtained through NGS. Artifacts generated by the equipment during the method are shown in orange, a length heteroplasmy not reported by the manufacturer is shown in blue, and positions whose polymorphisms were not detected during sequencing are shown in red.

| C1 (20 pg/ µL) | | | C2 (5 pg/ µL) | | | C3 (1 pg/ µL) | | | C4 (0.25 pg/ µL) | | |
|---|---|---|---|---|---|---|---|---|---|---|---|
| **R1** | **R2** | **R3** | **R1** | **R2** | **R3** | **R1** | **R2** | **R3** | **R1** | **R2** | **R3** |
| 73G | 73G | 73G | 73G | 73G | 73G | 73G | 73G | 73G | 73G | 73G | 73G |
| 152C | 152C | 152C | 152C | 152C | 152C | 152C | 152C | 152C | 152C | 152C | 152C |
| 199C | 199C | 199C | 199C | 199C | 199C | 199C | 199C | 199C | 199C | 199C | 199C |
| 204C | 204C | 204C | 204C | 204C | 204C | 204C | 204C | 204C | 204C | 204C | 204C |
| 207A | 207A | 207A | 207A | 207A | 207A | 207A | 207A | 207A | 207A | 207A | 207A |
| | 249del | | | | | | | | | | 249del |
| 250C | 250C | 250C | 250C | 250C | 250C | 250C | 250C | 250C | 250C | 250C | 250C |
| 263G | 263G | 263G | 263G | 263G | 263G | 263G | 263G | 263G | 263G | 263G | 263G |
| | | | | | 309del | | | 309del | | | |
| 315.1C | 315.1C | 315.1C | 315.1C | 315.1C | 315.1C | 315.1C | 315.1C | 315.1C | 315.1C | 315.1C | 315.1C |
| 455Y/del | 455del | | 455del | 455Y/del | 455del | 455Y/del | 455Y/del | | | | |
| 460C | 460C | 460C | 460C | 460C | 460C | 460C | 460C | 460C | 460C | 460C | 460C |
| 573.4C | 573.4C | 573.4C | 573.4C | 573.4C | 573.4C | 573.4C | 573.4C | 573.4C | 573.4C | 573.4C | |
| 16129A | 16129A | 16129A | 16129A | 16129A | 16129A | 16129A | 16129A | 16129A | 16129A | 16129A | 16129A |
| 16223T | 16223T | 16223T | 16223T | 16223T | 16223T | 16223T | 16223T | 16223T | 16223T | 16223T | 16223T |
| 16391A | 16391A | 16391A | 16391A | 16391A | 16391A | 16391A | 16391A | 16391A | 16391A | 16391A | 16391A |
| 16519C | 16519C | 16519C | 16519C | 16519C | 16519C | 16519C | 16519C | 16519C | 16519C | 16519C | 16519C |

**Table S13:** RS1 sequences obtained through NGS. Artifacts generated by the equipment during the method are shown in orange.

| C1 (20 pg/ µL) | | | C2 (5 pg/ µL) | | | C3 (1 pg/ µL) | | | C4 (0.25 pg/ µL) | | |
|---|---|---|---|---|---|---|---|---|---|---|---|
| **R1** | **R2** | **R3** | **R1** | **R2** | **R3** | **R1** | **R2** | **R3** | **R1** | **R2** | **R3** |
| 73G | 73G | 73G | 73G | 73G | 73G | 73G | 73G | 73G | 73G | 73G | 73G |
| 150T | 150T | 150T | 150T | 150T | 150T | 150T | 150T | 150T | 150T | 150T | 150T |
| 152C | 152C | 152C | 152C | 152C | 152C | 152C | 152C | 152C | 152C | 152C | 152C |
| 263G | 263G | 263G | 263G | 263G | 263G | 263G | 263G | 263G | 263G | 263G | 263G |
| 295T | 295T | 295T | 295T | 295T | 295T | 295T | 295T | 295T | 295T | 295T | 295T |
| | | | | | 309del | | | | | | 309del |
| 315.1C | 315.1C | 315.1C | 315.1C | 315.1C | 315.1C | 315.1C | 315.1C | 315.1C | 315.1C | 315.1C | 315.1C |
| | | | | | | | | | 438del | | |
| 489C | 489C | 489C | 489C | 489C | 489C | 489C | 489C | 489C | 489C | 489C | 489C |
| 16069T | 16069T | 16069T | 16069T | 16069T | 16069T | 16069T | 16069T | 16069T | 16069T | 16069T | 16069T |
| 16126C | 16126C | 16126C | 16126C | 16126C | 16126C | 16126C | 16126C | 16126C | 16126C | 16126C | 16126C |
| 16193T | 16193T | 16193T | 16193T | 16193T | 16193T | 16193T | 16193T | 16193T | 16193T | 16193T | 16193T |
| 16278T | 16278T | 16278T | 16278T | 16278T | 16278T | 16278T | 16278T | 16278T | 16278T | 16278T | 16278T |

**Table S14:** HR1 sequences obtained through NGS. Artifacts generated by the equipment during the method are shown in orange, polymorphisms that have not been correctly determined are shown in blue, and positions whose polymorphisms were not detected during sequencing are shown in red.

| C1 (20 pg/ µL) | | | C2 (5 pg/ µL) | | | C3 (1 pg/ µL) | | | C4 (0.25 pg/ µL) | | |
|---|---|---|---|---|---|---|---|---|---|---|---|
| **R1** | **R2** | **R3** | **R1** | **R2** | **R3** | **R1** | **R2** | **R3** | **R1** | **R2** | **R3** |
| 73G | 73G | 73G | 73G | 73G | 73G | 73G | 73G | 73G | 73G | 73G | 73G |
| 150T | 150T | 150T | 150T | 150T | 150T | 150Y | 150T | 150Y | 150T | 150T | 150T |
| 152C | 152C | 152C | 152C | 152C | 152C | 152C | 152C | 152Y | 152C | 152C | 152Y |
| 263G | 263G | 263G | 263G | 263G | 263G | 263G | 185R | 185R | 263G | 263G | 263G |
| 295T | 295T | 295T | 295T | 295T | 295T/del | 295T | 188R | 188R | 295T | 295T | 295T |
| 315.1C | 315.1C | 315.1C | 315.1C | 315.1C | 309del | 315.1C | 228R | 228R | 315.1C | 309del | 315.1C |
| 489C | 489C | 489C | 489C | 489C | 315.1C | 489C | 263G | 263G | 489C | 315.1C | 489C |
| 16069T | 16069T | 16069T | 16069T | 16069T | 489C | 16069T | 295T | 295T | 16069Y | 489C | 16069T |
| 16126C | 16126C | 16126C | 16126C | 16126C | 16069T | 16126C | 315.1C | 309del | 16126Y | 16069T | 16126C |
| 16193T | 16193T | 16193T | 16193T | 16193T | 16126C | 16150Y | 462Y | 315.1C | 16193T | 16126C | 16193T |
| 16278T | 16278T | 16278T | 16278T | 16278T | 16193T | 16193Y | 489C | 361del | 16278T | 16193T | 16278Y |
| 16488T | 16488T | 16488T | 16488T | 16488T | 16278Y | 16278Y | 16069T | 489C | 16488Y | 16278T | 16488Y |
| | | | | | 16488T | 16488Y | 16126C | 16069T | 16519C | 16488T | 16519Y |
| | | | | | | 16519Y | 16150Y | 16126C | | | |
| | | | | | | | 16193T | 16150Y | | | |
| | | | | | | | 16278T | 16193Y | | | |
| | | | | | | | 16390R | 16278Y | | | |
| | | | | | | | 16488T | 16488T | | | |
| | | | | | | | 16519Y | | | | |

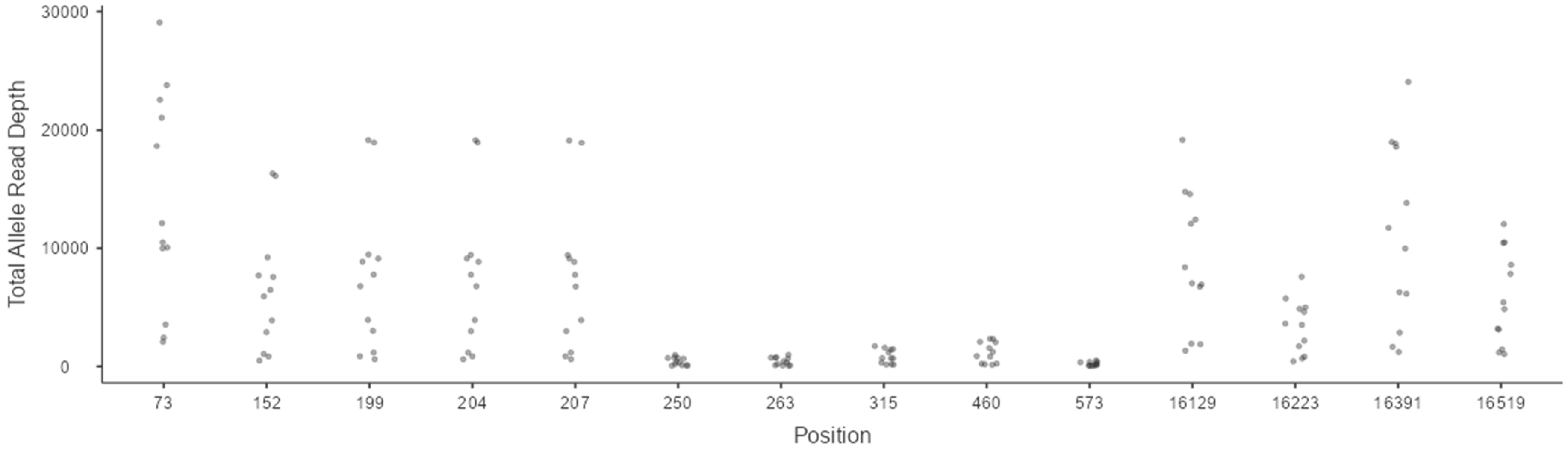


**Figure S1:** Jitter plot showing the total allele read depth of the expected polymorphisms across all concentrations and replicates for sample C007.

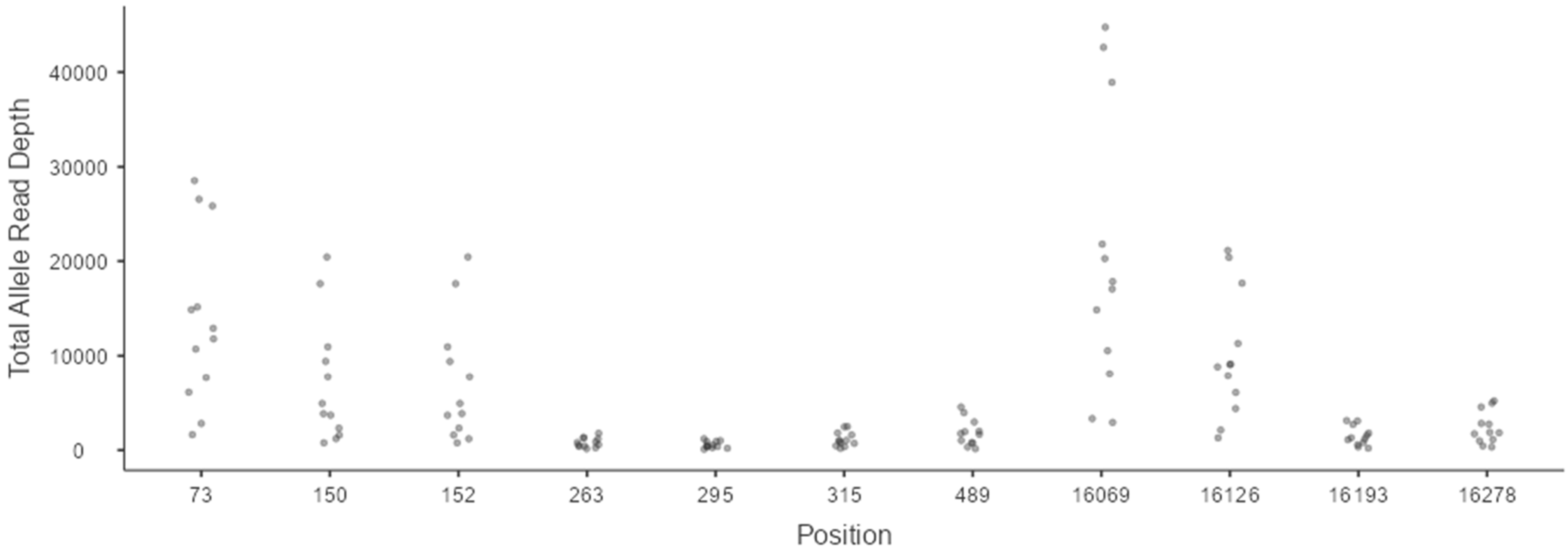


**Figure S2:** Jitter plot showing the total allele read depth of the expected polymorphisms across all concentrations and replicates for sample RS1.

**Table S15:** Sequences obtained via NGS.

| Sample | Concordance | Analyst | Sequence |
| --- | --- | --- | --- |
| RS4 | 71.4% | A1 | 263G, 309.2C*, 315.1C, 438del*, 456T, 16304C, 16519C |
| | | A2 | 263G, 309.1C*, 315.1C, 456T, 16304C, 16519C |
| RS5 | 85.7% | A1 | 73G, 152C, 199C, 250C, 263G, 315.1C, 494A, 573.4C*, 16129A, 16148T, 16223T, 16391A, 16519C |
| | | A2 | 73G, 152C, 199C, 250C, 263G, 309.1C*, 315.1C, 494A, 573.5C*, 16129A, 16148T, 16223T, 16391A, 16519C |
| RS8 | 87.5% | A1 | 73G, 263G, 315.1C, 497T, 16224C, 16311C, 16519C |
| | | A2 | 73G, 263G, 309del*, 315.1C, 497T, 16224C, 16311C, 16519C |
| RS9 | 62.5% | A1 | 263G, 309.1C, 315.1C, 16181M*, 16183M*, 16189C, 16193del*, 16298C |
| | | A2 | 263G, 309.1C, 315.1C, 16183C*, 16189C, 16193.1C*, 16298C |

* Discrepancies between runs.
